# Higher integrity of the motor and visual pathways in long-term video game players

**DOI:** 10.3389/fnhum.2015.00098

**Published:** 2015-03-10

**Authors:** Yang Zhang, Guijin Du, Yongxin Yang, Wen Qin, Xiaodong Li, Quan Zhang

**Affiliations:** ^1^Department of Radiology and Tianjin Key Laboratory of Functional Imaging, Tianjin Medical University General Hospital Tianjin, China; ^2^Department of Radiology, Linyi People’s Hospital Linyi, China; ^3^Department of Psychology, Linyi Fourth People’s Hospital Linyi, China

**Keywords:** diffusion tensor imaging, inferior fronto-occipital fasciculus, inferior longitudinal fasciculus, superior longitudinal fasciculus, video game, white matter

## Abstract

Long term video game players (VGPs) exhibit superior visual and motor skills compared with non-video game control subjects (NVGCs). However, the neural basis underlying the enhanced behavioral performance remains largely unknown. To clarify this issue, the present study compared the whiter matter integrity within the corticospinal tracts (CST), the superior longitudinal fasciculus (SLF), the inferior longitudinal fasciculus (ILF), and the inferior fronto-occipital fasciculus (IFOF) between the VGPs and the NVGCs using diffusion tensor imaging. Compared with the NVGCs, voxel-wise comparisons revealed significantly higher fractional anisotropy (FA) values in some regions within the left CST, left SLF, bilateral ILF, and IFOF in VGPs. Furthermore, higher FA values in the left CST at the level of cerebral peduncle predicted a faster response in visual attention tasks. These results suggest that higher white matter integrity in the motor and higher-tier visual pathways is associated with long-term video game playing, which may contribute to the understanding on how video game play influences motor and visual performance.

## INTRODUCTION

Video game playing has become ubiquitous entertainment today and is particularly popular among the youth. Behavioral studies have reported that video gaming may have favorable effects on visual related skills (such as attention, memory, and spatial discrimination; Green and Bavelier, 2003, 2006a,b, 2007; Riesenhuber, 2004; Castel et al., 2005; Donohue et al., 2010; Bavelier et al., 2012) and motor skills (Schlickum et al., 2009; Lynch et al., 2010). Besides, long-term video game play can also drive extensive structural reorganization of the brain. For example, increased gray matter volumes have been detected in the right posterior parietal cortex (Tanaka et al., 2013), the right hippocampal formation, the right dorsolateral prefrontal cortex, the bilateral cerebellum (Kuhn et al., 2014a), and the left striatum (Kuhn et al., 2011) in video game players (VGPs). Additionally, it is reported that the duration of video game play is significantly associated with the gray matter volume of the entorhinal cortex, hippocampus occipital lobe (Assaf and Pasternak, 2008), and the thickness of the left frontal cortex (Kuhn et al., 2014b). In addition to these positive effects, the duration of action-based role-playing is associated with atrophy of the entorhinal cortex (Kuhn and Gallinat, 2013). However, studies focused on the reorganization of white matter (WM) pathway in VGPs remains scarce.

Motor skill training-induced experience-dependent changes in WM microstructure have been reported in pianists (Bengtsson et al., 2005) and jugglers (Scholz et al., 2009). These studies demonstrate increased fractional anisotropy (FA) values in the right internal capsule and the right posterior intraparietal sulcus, respectively. In addition, increased FA values have also been detected in the WM of frontal, cingulate and striato-thalamic areas that are related to attentional control, working memory, executive regulation, and problem solving in long-term trained Baduk players (Lee et al., 2010). All the aforementioned studies have demonstrated the association between specific training and structural plasticity in related WM pathways.

It is well known that video games require intense processing of visual stimuli, and complex motor demands. Participants are required to concentrate on these stimuli rapidly process the related information, and respond quickly by manipulating the keyboard/mouse using their fingers. Thus, long-term video game play can be considered intense training of visual processing and finger skills. Sensorimotor information is primarily conveyed by corticospinal tracts (CST) that are critically important for independent finger movements in humans. Higher visual process (e.g., visual attention, visual memory, visual imagining) are associated with WM pathways including the superior longitudinal fasciculus (SLF; Umarova et al., 2010; Chechlacz et al., 2012; Klarborg et al., 2013; Parks and Madden, 2013), inferior longitudinal fasciculus (ILF; Chechlacz et al., 2012; Parks and Madden, 2013), and inferior fronto-occipital fasciculus (IFOF; Umarova et al., 2010; Chechlacz et al., 2012). Therefore, it is plausible to hypothesize that the WM integrity of the motor and visual pathways may increase in VGPs as a consequence of long-term video game play.

In the current study, diffusion tensor imaging (DTI) was performed in adolescent VGPs and non-video game control subjects (NVGCs), and voxel-based intergroup comparisons of the FA values were conducted to investigate the possible influence of the long-term video game play on the microstructure of the CST, SLF, ILF, and IFOF in adolescents. We hypothesized that the WM integrity (represented as FA values) of both the motor and visual pathways would be higher in VGPs compared to NVGCs. We also predicted that higher WM integrity would predict superior visual attentional performance in the VGP group.

## MATERIALS AND METHODS

### SUBJECTS

Forty-five right-handed male subjects were recruited from the Health School of Linyi, Shandong Province. Only males underwent testing because of the relatively small number of females with long-term video game experience. Video game experience was assessed via a self-report questionnaire that questioned about the length and amount of experience across several video game genres (the detailed information is listed in **Table 1**). VGPs were defined as subjects who had played video games for more than 6 months with a frequency of at least 10 h per week. Twenty-eight subjects (mean age = 16.9, SD = 2.2) were included in this category (VGP mean = 19 h per week). NVGCs were defined as the subjects who had little to no video game experience. Seventeen subjects (mean age = 17.1, SD = 1.3) fell into this category. Thirteen subjects had never played video games and the other four subjects had played video games with a frequency of 1–2 h per week. All subjects had no experience with special motor skills, such as piano playing or table tennis.

**Table 1:** Game genres and duration in video game players.

Subject ID	Age (year)	Game duration(Month)	Game time per week (hour)	Type of game
1	17	24	17	Audition dance battle online
2	23	48	23	Warcraft
3	18	8	24	Cross fire
4	14	24	19	Cross fire
5	14	5	13	Audition dance battle online
6	16	24	23	Need for speed
7	16	24	19	Need for speed
8	16	26	23	Need for speed
9	18	60	23	Cross fire
10	15	24	23	Need for speed
11	17	6	15	Need for speed
12	17	60	20	Need for speed
13	18	60	24	Need for speed
14	22	24	13	Need For speed
15	17	36	15	Cross fire
16	15	24	15	Audition dance battle online
17	14	72	15	Cross fire
18	16	18	13	Audition dance battle online
19	17	36	27	Audition dance battle online
20	15	36	27	Cross fire
21	20	6	23	Warcraft
22	18	48	23	Cross fire
23	18	24	12	Need for speed
24	19	60	23	Warcraft
25	17	48	15	Need for speed
26	18	12	25	Warcraft
27	15	48	13	Cross fire
28	14	8	16	Warcraft


In addition, the exclusion criteria for all subjects were magnetic resonance imaging (MRI) contraindications, visible abnormalities on conventional MRI, mental disorders, neurological diseases, or any clinically relevant abnormalities such as insomnia, migraines, tinnitus, and substance abuse, or dependence. This study was approved by the Ethical Committee of Tianjin Medical University General Hospital and written informed consent was obtained from all subjects or their guardians. All subjects received medical reference books as compensation.

### BEHAVIORAL TASK

**FIGURE 1:**
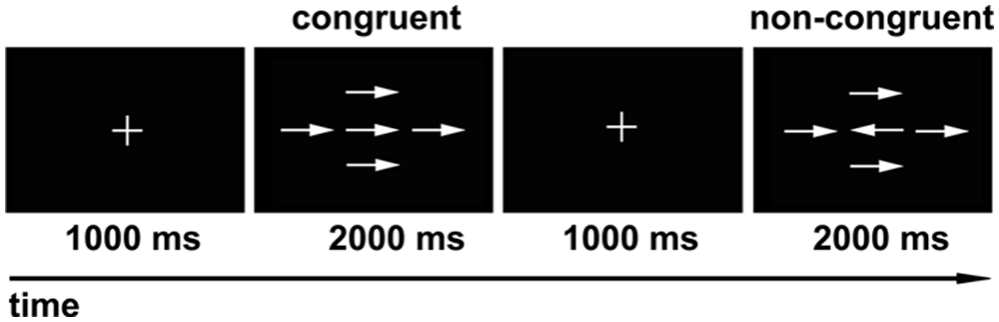
**Modified version of visual attention network test (ANT).** During each trial, the “+” was presented in the center of the screen for 1000 ms first, and then the arrow symbols showed for 2000 ms. The subjects were asked to determine the direction of the central arrow regardless of the surrounding four arrows. The numbers of central arrow toward the left and right were equal, and the four surrounding arrows oriented equally in the congruent or non-congruent direction with the central arrow.

We used a modified version of the Attention Network Test (ANT) described by Fan et al. (2002) as our behavioral task (**Figure 1**). Eprime software (Psychology Software Tools, Pittsburgh, PA, USA) was used for visual stimulus presentation and for participant response recording. The experiment consisted of 80 trials presented in a random order. Each trial included two stages. The first stage was the fixed stage with a “+” in the center of the screen, and the second stage was the test stage, which consisted of a central target arrow symbol and four surrounding arrow symbols with the same direction (congruent), or inverse direction (non-congruent) relative to the central arrow. During each trial, the subjects were asked to fixate on the “+” for 1000 ms. Then the “+” was replaced by arrow symbols for 2000 ms. The subjects were asked to determine the direction of the central arrow while ignoring the surrounding four arrow symbols, and respond as fast as possible by clicking the left (left direction), or right key (right direction) of the mouse. If the subjects reacted within 2000 ms, the presentation would go directly to the next trial; if no response was made, the presentation would go to the next trial after 2000 ms. The total number of central arrows towards the left and right was equal, and the four surrounding arrows were oriented equally in the congruent or non-congruent directions with the central arrow. The formal experiment was performed after the subjects have learned and practiced the task. The average response times and accurate rates were calculated individually for all subjects.

### MRI ACQUISITION

Magnetic resonance images were obtained on a 1.5 Tesla scanner. DTI was performed using a single-shot spin-echo echo planar imaging sequence with the following parameters: repetition time = 8300 ms, echo time = 80.1 ms, flip angle = 90^∘^, matrix = 128 × 128, field of view = 25.6 cm × 25.6 cm, number of excitation = 2, slice thickness = 5 mm, 25 slices with no gap, voxel size = 2 mm × 2 mm × 5 mm. The diffusion-sensitizing gradients were applied along 13 non-collinear directions with a b value of 1000 s/mm^2^, and one volume was also acquired without diffusion weighting (*b* = 0).

### DTI DATA PROCESSING

All diffusion-weighted images were visually inspected by two radiologists for apparent artifacts due to subject motion and instrument malfunction. Eddy current distortions and head motion artifacts in the DTI dataset were corrected by applying affine alignment of each diffusion-weighted image to the non-diffusion image, using the FMRIB’s diffusion toolbox (FSL ^1^). The robust brain extraction tool (BET) was used to extract the brain for each participant’s DTI images. Following brain extraction, all eigenvalues, and FA maps were calculated using a DTIFit within the FMRIB diffusion toolbox in FSL. Each subject’s FA map was normalized to the Montreal Neurological Institute (MNI) space ^2^ with a resolution of 3 mm × 3 mm × 3 mm, by utilizing the Statistical Parametric Mapping software (SPM8 ^3^). Further, all normalized FA images were spatially smoothed by an isotropic full-width at the half maximum Gaussian kernel of 6 mm × 6 mm × 6 mm.

**FIGURE 2:**
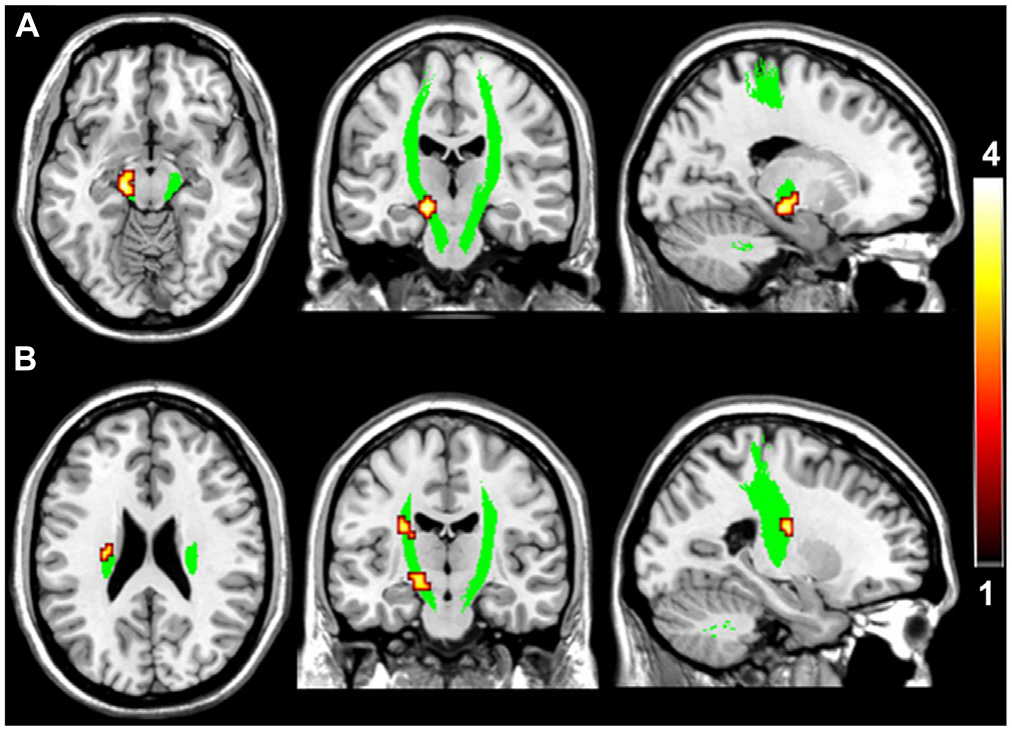
**Brain regions with higher fractional anisotropy (FA) in the motor pathway in VGPs.** The area in green is the WM mask of bilateral CST. Areas in red to yellow at midbrain **(A)** and ventricular body level **(B)** of the left CST are regions where FA values were significantly higher in VGPs relative to NVGCs (*P* < 0.01, corrected).

**FIGURE 3:**
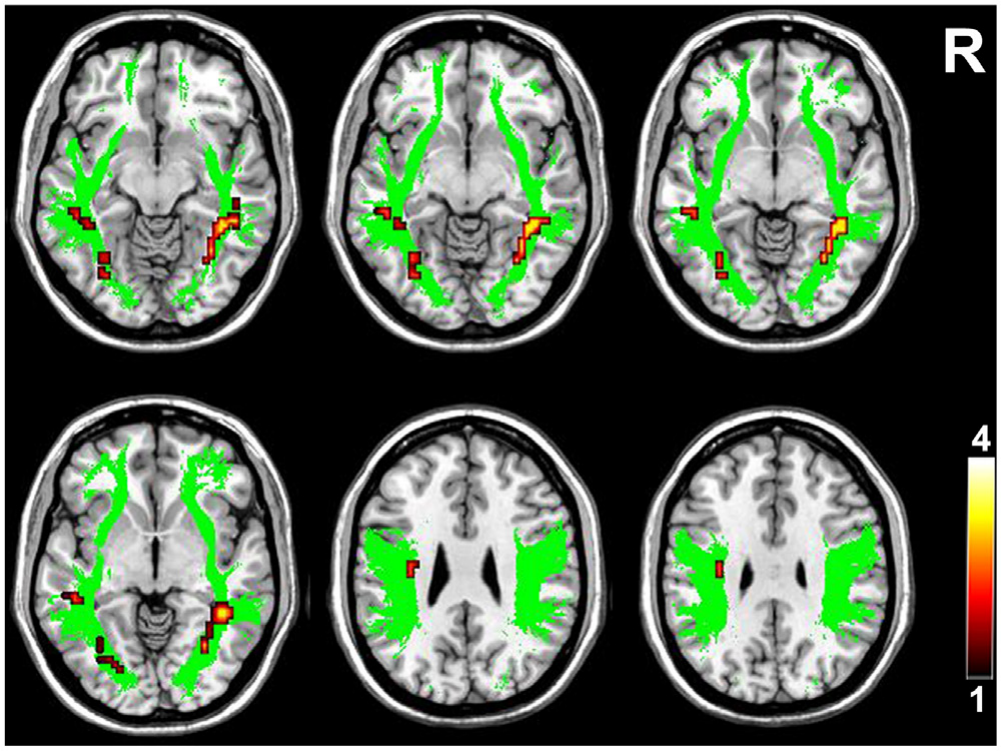
**Brain regions with higher FA in the higher-tier visual pathway in VGPs.** Area in green is the WM mask of higher-tier visual pathway including the bilateral SLF, ILF, and IFOF. Areas in red to yellow are regions where FA values were significantly higher in VGPs relative to NVGCs (*P* < 0.01, corrected). R, right hemisphere.

### STATISTICAL ANALYSES.

Intergroup differences in age, education, and behavioral performance (including response times and accurate rates) were compared using a two-sample *t*-test with SPSS (SPSS18.0, Chicago, IL, USA). The significance level was set at *P* < 0.05.

Voxel-wise intergroup comparisons of the FA values were performed using two-sample *t*-test within the two masks of the WM pathways, namely the motor pathway including the bilateral CST (**Figure 2**), and the visual pathway including the bilateral SLF, ILF, and IFOF (**Figure 3**). Each mask contained equivalent anatomical areas in the bilateral hemispheres and was derived from the Johns Hopkins University (JHU) WM tractography atlas provided by the FSL software distribution (Wakana et al., 2004). A threshold of 10% was applied to exclude low probability voxels. Because age may be associated with the WM integrity during development, age was treated as a covariate of no interest in the voxel-wise statistical analysis. A correction for multiple comparisons was performed using the Monte Carlo simulation, resulting in a corrected threshold of *P* < 0.05 (AlphaSim program, parameters including: single voxel *P* = 0.01, 1000 simulations, full width at half maximum = 6 mm, cluster connection radius *r* = 5 mm, and the mask of CST and combined SLF, ILF, and IFOF, respectively). The cluster threshold was 8 and 15 voxels for CST and the combined SLF, ILF, and IFOF, respectively.

The regions with significant intergroup differences in FA values detected with the voxel-wise analysis were defined as ROIs. Partial correlation analyses controlling for age were performed between the averaged FA values within the ROIs and the video gaming duration and the behavioral performance in VGPs. The significance level was set at *P* < 0.05.

## RESULT

### DEMOGRAPHIC AND BEHAVIORAL STATISTICS

There were no significant intergroup differences in age, education, and accuracy rate of the behavioral task. The response times of the visual attention task were significantly faster in VGPs than NVGCs (*P* < 0.05). The intergroup comparisons of demographic and behavioral performance are listed in **Table 2**.

**Table 2:** Demographic characteristics and behavioral performance of subjects.

	VGPs (*n* = 28)	NVGCs (*n* = 17)	*t*	*P*
Age (years)	16.9 ± 2.2	17.1 ± 1.3	0.361	0.720
Education (years)	9.9 ± 1.8	10.0 ± 0.4	0.235	0.815
RT (ms)	619.2 ± 84.8	690.3 ± 119.9	2.351	0.023*
ACC (%)	97.1 ± 3.0	98.2 ± 2.2	1.364	0.180

*Two-sample two-tailed *t*-tests. Significant level is set as *P* < 0.05. ACC, accurate of attention network test; NVGCs, non-video-game controls; RT, response time; VGPs, video-game players.

**Table 3:** The regions with higher FA values in VGPs than in NVGCs.

Fiber tracts	Regions	Cluster size	MNI coordinates			*t*


		(voxel)	*X*	*Y*	*Z*	
Left CST	Cerebral peduncle level	36	-12	-24	-9	-3.137
Left CST	Ventricular body level	11	-24	-12	21	-1.042
Left SLF	Frontal region	25	-30	-15	24	-1.957
Left ILF/IFOF	Temporal region	17	-48	-36	-3	-5.421
Left ILF/IFOF	Occipital region	20	-24	-75	0	-5.421
Right ILF/IFOF	Temporal region	74	33	-51	-9	-5.881

*IFOF, inferior fronto-occipito fasciculus; ILF, inferior longitudinal fascicle; MNI, Montreal Neurological Institute; NVGCs, non-video-game controls; SLF, superior longitudinal fascicle; VGPs, video-game players.*

**FIGURE 4:**
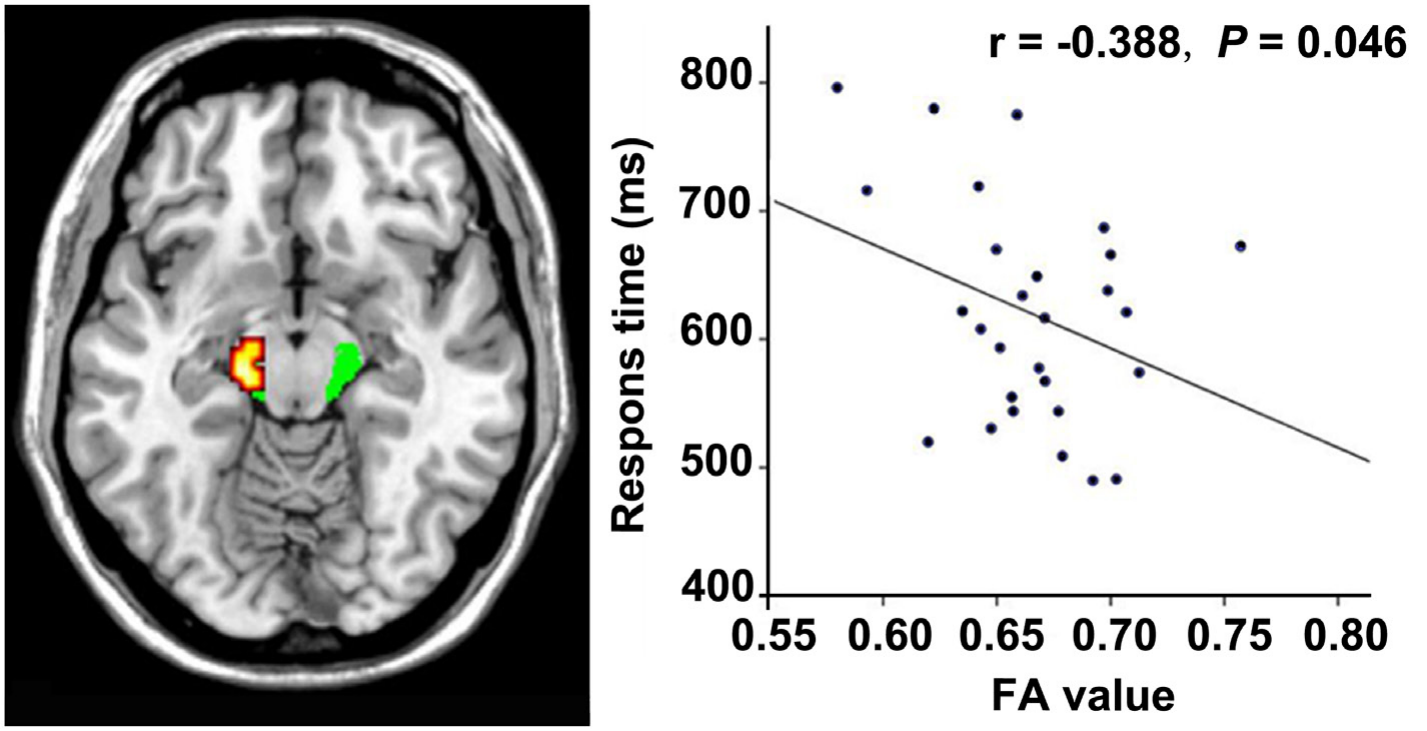
**Correlation between FA and response time within the VGPs.** FA values in the left CST at cerebral peduncle level correlates negatively with the response time of visual attentional task within the VGPs after controlling for age (*r* = -0.388, *P* = 0.046).

### INTERGROUP DIFFERENCES IN FA VALUES

Compared with NVGCs, the FA values at the lateral ventricle body and cerebral peduncle level of the left CST were significantly higher in VGPs (*P* < 0.01, corrected; **Figure 2**). There were four clusters within the visual pathway that demonstrated significantly higher FA values in VGPs than in NVGCs (*P* < 0.01, corrected; **Figure 3**), including one cluster located in the frontal region of the left SLF, two clusters located in the temporal and occipital region of the left ILF/IFOF; and one cluster located in the temporal region of the right ILF/IFOF. The MNI coordinates of the aforementioned six clusters are lsited in **Table 3**. No region exhibited significantly lower FA values in VGPs than in NVGCs.

### CORRELATIONS BETWEEN FA VALUE AND BEHAVIORAL PERFORMANCE

In the VGPs, there was a significant negative correlation between the response time of the visual attention task and the FA value in the ROI at the midbrain level of the left CST after controlling for age (*r* = -0.388, *P* = 0.046; **Figure 4**). There were no significant associations between the FA values in the other ROIs and the behavioral performance or between the FA values in all ROIs, and video gaming duration in the VGPs.

## DISCUSSION

In the present study, DTI-derived FA values were used to investigate the reorganization of WM integrity in the motor and visual pathways in adolescent VGPs. We found higher WM integrity in the CST, SLF, ILF, and IFOF in VGPs compared to the age- and education-matched NVGCs. We also found that higher FA values in the left CST at the level of the cerebral peduncle predicted faster respond speeds for the visual attentional task. This study may be helpful for understanding the mechanism through which extensive video game play influences motor and visual performance.

### FA AND WM PLASTICITY

The FA index is the most widely used parameter of DTI. The FA value represents the motional anisotropy of water molecules, and is sensitive to the presence and integrity of WM fibers (Assaf and Pasternak, 2008). Changes in several features of the WM, such as axon myelination, axon diameter, the axon membrane, axon transport, neurofibrils, and packing density, could have an effect on the WM integrity and is represented as the FA value (Beaulieu, 2002).

Long-term exposure to special stimulation may induce plasticity that provides the nervous system with the capacity to modify its organization and structure to adapt to the environment (Nathan et al., 2011). Plasticity in WM mainly encompasses myelination of previously unmyelinated axons and the remodeling of existing myelin sheaths (Boulanger and Messier, 2014). Training can induce WM plasticity if it occurs during a period when the involved fiber tracts are still maturing (Bengtsson et al., 2005). WM maturation is a process of continuous myelination, and continues at least until the young adulthood. In the present study, all the VGPs enrolled were adolescents with myelin sheaths still under maturation. It is plausible to believe that developing WM fiber tracts in adolescent VGPs may be more susceptible to long-term video game stimuli and more prone to plasticity. An experiment in mice has demonstrated that myelination can be stimulated by electrical activity in pre-myelinated axons (Demerens et al., 1996).

In addition to the degree of myelination, other mechanisms including the number of myelinated axons in a tract, axon diameters have been reported to contribute to increased functional performance with learning (Zatorre et al., 2012). Long-term exposure to video games could be considered receiving intense training of the finger-motor and visual skills. As a result, integrity in these WM fiber tracks might be promoted leading to higher FA values in VGPs.

### HIGHER FA VALUES OF THE MOTOR PATHWAYS IN VGPS

The CST contains descending fibers from the primary sensorimotor and premotor cortices; conveys sensorimotor information, and is critically important for independent finger movements (Phillips and Porter, 1977; Porter, 1985). In this study, FA values of the left CST were higher in VGPs than in NVGCs. All the VGPs recruited in this study were right-handed and their right hands were used more frequently than their left hands when playing video games. The stronger sensorimotor demand for the right hand made the left sensorimotor pathway obtain more activity and lead to higher integrity of the left CST in VGPs. This type of long-term motor-training related plasticity of the CST has also been reported in other motor-training related studies [demonstrating increased FA values of CST in jugglers (Scholz et al., 2009) and pianists (Bengtsson et al., 2005)]. Furthermore, the results of a correlation analysis demonstrated that FA values in the midbrain level of the left CST were negatively correlated with RT. These results might suggest a close relationship between long-term video game play and the higher integrity of the left CST in VGPs.

### HIGHER FA VALUES OF THE VISUAL PATHWAYS IN VGPS

The SLF is the primary and direct pathway providing bidirectional information transfer between the frontal and parietal cortices (Petrides and Pandya, 1984; Schmahmann and Pandya, 2009). It is related to the higher visual processing, especially for visuospatial attention ( Umarova et al., 2010; Chechlacz et al., 2012; Klarborg et al., 2013; Parks and Madden, 2013). The ILF reciprocally connects the temporal and occipital cortices and it appears to mediate the fast transfer of visual signals to the anterior temporal regions and neuromodulatory back-projections from the amygdala to primary visual areas (Catani et al., 2003). The ILF is involved in the face recognition (Fox et al., 2008) and visual memory (Ross, 2008). The IFOF connects the frontal and the occipital cortices (Koch et al., 2014) and is likely to play an important role in visual processing (Fox et al., 2008), attention (Doricchi et al., 2008), and facial emotion recognition (Philippi et al., 2009).

The SLF, ILF, and IFOF are common WM pathways supporting interactions across different cortical regions related to higher visual processing. The association between the integrity of SLF, ILF, and IFOF and visual behavioral performance has already been demonstrated in many studies ( Umarova et al., 2010; de Schotten et al., 2011; Chechlacz et al., 2012; Klarborg et al., 2013; Mayer and Vuong, 2013; Parks and Madden, 2013). Decreased integrity in the SLF (Shinoura et al., 2009), ILF (Konrad et al., 2012), and IFOF (Aralasmak et al., 2006) is associated with a variety of visual cognitive functional deficits. In a study on simultanagnosia, Chechlacz et al. (2012) proposed that extensive disconnection within the visuospatial attention related WM pathways (SLF, ILF, and IFOF) partially contributed to the slowed processing and poor awareness of multiple stimuli. As for the VGPs in our study, long-term training with complex visual scenes in the video games might enhance their visual skills related to visual processing and their awareness of multiple stimuli. Previous studies have already demonstrated that action games can modify visual processing (see a review of Riesenhuber, 2004). The experiments performed by Green and Bavelier (2003, 2006a,b, 2007) have also suggested that video game experience could enhance the capacity of players’ visual attention. Thus, it is plausible to presume that the higher integrity of the SLF, ILF, and IFOF in our VGPs might be derived from the plasticity induced by long-term video game play.

The response times in our behavioral task were shorter in VGPs than in NVGCs and were negatively correlated to the FA value of the left CST at the midbrain level, but were not correlated to the FA values of all ROIs in the SLF, ILF, and IFOF in VGPs. A possible explanation might be that the increased behavioral ability was primarily attributed to the fast finger responses rather than increased visual processing capacity. Another possible explanation is that higher FA values of SLF, ILF, and IFOF derived from multiple aspects of video game related visual training might not present with significant correlations with any single aspect of visual capacity, such as visual attention capacity tested in our study.

There are several limitations in our study that should be mentioned. First, the sample size in this study was relatively small, which might reduce the power. Second, only the visual attention capacity was tested in this study, which cannot cover all aspects of video game related behavioral performance. Third, only male adolescents were recruited in the present study. We cannot determine whether these results could extend to females or other age groups. Finally, it should be noted that, with this cross-sectional study, we cannot determine whether higher FA values are a consequence of video game playing or represent *a priori* characteristics that lead to better performance (and therefore more willingness to play video games). In addition, if the higher FA values were video game playing-induced, the possibility of long-term adverse effects of video game play on the WM integrity cannot be excluded. A longitudinal study may be helpful to determine the causal relationship between higher FA values and video game play, and to evaluate the long-term effects of video game playing on WM integrity.

## CONCLUSION

In conclusion, we used DTI to investigate WM integrity among male adolescent VGPs. VGPs had significantly higher FA values in the left CST, SLF, bilateral ILFs, and IFOFs. Faster responses were associated with higher FA values in the left CST in the VGPs independent of age. These observations demonstrated that higher WM integrity in the motor and higher-tier visual pathways is associated with long-term video game playing, which may contribute to the understanding of how extensive video game play may alter motor and visual performance.

## AUTHOR CONTRIBUTIONS

YZ, XL, and QZ designed research; YZ, GD, YY, WQ, and QZ performed research; GD, YY and XL were involved in the clinical assessment; YZ, QZ, GD, and WQ analyzed data; YZ, QZ, GD, and XL wrote the paper.

## ACKNOWLEDGMENT

The authors wish to thank all the subjects for their participation in the study.

## Conflict of Interest Statement

The authors declare that the research was conducted in the absence of any commercial or financial relationships that could be construed as a potential conflict of interest.

## Footnotes

1 http://www.fmrib.ox.ac.uk/fsl

2 http://www.mni.mcgill.ca/

3 http://www.fil.ion.ucl.ac.uk/spm/software/spm8
