# Corrigendum: Higher integrity of the motor and visual pathways in long-term video game players

**DOI:** 10.3389/fnhum.2019.00125

**Published:** 2019-04-09

**Authors:** Yang Zhang, Guijin Du, Yongxin Yang, Wen Qin, Xiaodong Li, Quan Zhang

**Affiliations:** ^1^Department of Radiology and Tianjin Key Laboratory of Functional Imaging, Tianjin Medical University General Hospital, Tianjin, China; ^2^Department of Radiology, Linyi People's Hospital, Linyi, China; ^3^Department of Psychology, Linyi Fourth People's Hospital, Linyi, China

**Keywords:** diffusion tensor imaging, inferior fronto-occipital fasciculus, inferior longitudinal fasciculus, superior longitudinal fasciculus, video game, white matter

In the original article, there was an error. The statement of the written informed consent procedures was incorrect because of inappropriate wording.

A correction has been made to the **Materials and Methods**, subsection **Subjects**, paragraph two:

“This study was approved by the Ethical Committee of Tianjin Medical University General Hospital and written informed consent was obtained from all subjects or their guardians.”

The authors apologize for this error and state that this does not change the scientific conclusions of the article in any way. The original article has been updated.

